# Differential expression of topoisomerase IIα protein in salivary gland carcinomas: histogenetic and prognostic implications

**DOI:** 10.1186/1471-2407-9-72

**Published:** 2009-02-27

**Authors:** Shin-ichiro Maruya, Takashi Shirasaki, Takahiko Nagaki, Seiji Kakehata, Hidekachi Kurotaki, Hiroki Mizukami, Hideichi Shinkawa

**Affiliations:** 1 Department of Otolaryngology, Hirosaki University School of Medicine, Hirosaki, Japan; 2 Department of Head and Neck Oncology and Surgery, International University of Health and Welfare Mita Hospital, Tokyo, Japan; 3 Department of Pathology, Odate Municipal Hospital, Odate, Japan; 4 Department of Pathology, Hirosaki University School of Medicine, Hirosaki, Japan

## Abstract

**Background:**

Salivary gland carcinomas are relatively uncommon heterogeneous malignancies characterized by locoregional invasion and distant metastasis. Topoisomerase IIα (topoIIα), located at chromosome 17q21-22, is considered a major mediator of cell proliferation and DNA replication. The purpose of this study was to evaluate the expression of topoIIα in various types of salivary gland tumors and its biological significance.

**Methods:**

The protein expression of topoIIα was evaluated immunohistochemically in formalin-fixed, paraffin-embedded tissue from 54 salivary gland carcinomas and 20 benign tumors (10 pleomorphic adenomas and 10 Warthin's tumors). The primary salivary gland carcinoma specimens consisted of 17 adenoid cystic carcinomas, 7 adenocarcinomas not otherwise specified, 7 mucoepidermoid carcinomas, 6 salivary duct carcinomas, 3 acinic cell carcinomas, 3 carcinomas ex pleomorphic adenomas, 3 epithelial-myoepithelial carcinomas, 2 carcinosarcomas, 2 lymphoepithelial carcinomas, 2 myoepithelial carcinomas, 1 oncocytic carcinoma, and 1 squamous cell carcinoma. The associations between clinicopathological factors and outcome were analyzed.

**Results:**

Of the 54 primary salivary gland carcinomas, 38 (70%) showed positive expression (≥10%) of topoIIα protein, and 16 carcinomas (30%) and all benign tumors were negative (p < 0.001). Expression of topoIIα was more frequently observed in salivary duct carcinoma, carcinoma ex pleomorphic adenoma, adenocarcinoma, and adenoid cystic carcinoma, solid type, and it was associated with advanced stage and shortened survival.

**Conclusion:**

The results of the present study suggest that topoIIα expression is associated with histologically aggressive subtypes and shortened survival. Furthermore, it may provide useful prognostic information and suggests the potential efficacy of topoIIα-targeting therapy in patients with salivary gland carcinoma.

## Background

Salivary gland carcinomas are uncommon neoplasms, accounting for approximately 5% of those arising in the head and neck region [1]. These tumors are characterized by widely varied histological features with heterogeneous and unpredictable clinical behaviors. In general, these carcinomas may be categorized into low grade and high grade malignancies based on their origin from either the terminal (intercalated) or the excretory ductal epithelium, respectively [2]. Biological and clinical classifications have been dependent on this histogenetic classification, but they are occasionally diverse even within those categories [3]. Patients with higher histological grades appear to be more susceptible to locoregional recurrence and distant metastasis. However, effective treatment for unresectable tumors has not been established [4]. Although chemotherapy remains the main treatment modality for patients with advanced disease, the efficacy of systemic chemotherapy appears to be limited or controversial in high-grade salivary gland carcinomas.

Topoisomerase IIα (topoIIα), a nuclear enzyme, has a key role in DNA replication. Consistent with its major role in DNA replication, overexpression of topoIIα has been detected in cells with high proliferation activity [5,6]. The gene encoding topoIIα is located at chromosome 17q21-22 and encodes for a 170 kDa protein. Interestingly, this gene is also in close proximity to the HER2 oncogene, which encodes for a transmembrane tyrosine kinase receptor protein and has been shown to be frequently coamplified in several cancers, including breast cancer [7]. Overexpression of HER2 has been reported in salivary gland carcinomas of excretory cell origin, including salivary duct and squamous cell carcinomas, but not in those of intercalated cell origin, including adenoid cystic, acinic cell, and adenocarcinomas, and it is associated with poor clinical prognosis in patients with salivary duct carcinoma [8]. On the other hand, little information is available about the expression of topoIIα protein in salivary gland tumors.

TopoIIα is a known cellular target for anticancer agents such as anthracyclines. Anthracyclines inhibit the activity of topoIIα by stabilizing DNA cleavage, thus exerting anticancer effects [6]. Previous clinical investigations have shown that amplification and overexpression of topoIIα are associated with good response to anthracycline-based chemotherapy in several cancers, such as breast cancer [9-11]. Anthracyclines as a single agent or in combination therapy have shown certain effects in mucoepidermoid carcinomas and high-grade adenocarcinomas, whereas they likely have a limited effect in adenoid cystic carcinomas [4]. These observations suggest that identifying biological markers that can predict sensitivity to drugs may also allow stratification of patients for treatment. The purpose of the present study was to evaluate topoIIα expression levels in different histological types of salivary gland tumors and to estimate its potential use as a prognostic marker or clinical target in the treatment of patients with salivary gland cancers.

## Methods

### Tissue samples from surgical cases

Tumor specimens from 54 patients with primary salivary gland carcinomas who underwent surgery or biopsy between 1996 and 2007 were retrieved from the Department of Pathology, Hirosaki University, and associated hospitals. Tumor specimens consisted of 17 adenoid cystic carcinomas, 7 mucoepidermoid carcinomas, 7 adenocarcinomas not otherwise specified, 6 salivary duct carcinomas, 3 carcinomas ex pleomorphic adenomas, 3 acinic cell carcinomas, 3 epithelial-myoepithelial carcinomas, 2 myoepithelial carcinomas, 2 lymphoepithelial carcinomas, and 2 carcinosarcomas. There was only one sample of squamous cell carcinoma and one of oncocytic carcinoma. To compare the expression status of benign tumors, 10 pleomorphic adenomas and 10 Warthin's tumors were also selected (Table 1). All samples were fixed in a 10% formaldehyde solution and embedded in paraffin. Diagnosis and histological classification were based on the World Health Organization Classification (version 2005) [12]. Informed consents from patients were obtained for the use of resected tumor specimens. The study design and procedure involving the human tissue sampling collection were approved by the ethical board of Hirosaki University.

**Table 1:** TopoIIa expression status in different histologic types

	Topo IIα Expression Grade				
Histological type	0	1+	2+	3+	Total
*Benign tumor*					
Pleomorphic adenoma	10	0	0	0	10
Warthin's tumor	10	0	0	0	10
*Malignant tumor*					
Adenoid cystic carcinoma	5	7	3	2	17
Adenocarcinoma,	2	0	2	3	7
not otherwise specified					
Mucoepidermoid carcinoma	3	2	2	0	7
Salivary duct carcinoma	0	1	0	5	6
Acinic cell carcinoma	2	0	0	1	3
Carcinoma ex pleomorphc adenoma	0	0	0	3	3
Epithelial-myoepithelial carcinoma	2	1	0	0	3
Carcinosarcoma	0	0	1	1	2
Lymphoepithelial carcinoma	1	0	1	0	2
Myoepithelial carcinoma	0	1	0	1	2
Oncocytic carcinoma	1	0	0	0	1
Squamous cell carcinoma	0	0	0	1	1

### Immunohistochemistry

For immunohistochemistry, 4-μm-thick sections were deparaffinized in xylene and rinsed in ethanol. For antigen retrieval, the sections were heated for 15 min at 121°C in Target Retrieval Solution (DakoCytomation, Glostrup, Denmark) by autoclaving. Endogenous peroxidase was blocked by incubation in methanol with 0.3% H_2_O_2 _for 15 min, followed by rinsing in PBS containing 0.1% Tween 20. Immunohistochemical staining was performed with the EnVision™+ System Kit (DakoCytomation) according to the manufacturer's protocol. After treatment with blocking solution, the slides were incubated with the monoclonal primary antibody against topoisomerase IIα (clone Ki-S1; DakoCytomation; dilution 1:100) overnight at 4°C. The slides were then incubated at room temperature for 30 min with a dextran polymer conjugated with horseradish peroxidase enzyme and secondary anti-mouse antibody. The reaction products were detected with 3,3'-diaminobenzidine tetrahydrochloride as the chromogen, and the slides were counterstained with hematoxylin. The number of positive cells in 1000 tumor cells within 4–6 microscopic fields at ×200 magnification was counted and semiquantitatively graded as follows: 0 (negative, 0% to 9%), 1+ (focal, 10% to 24%), 2+ (moderate, 25% to 49%), and 3+ (diffuse, ≥50%). Quantitation of positive cells was conducted by 3 independent observers (S.M., H.M. and H.K.), and inter-observer concordance was over 95%.

### Statistical analysis

Statistical analysis was performed using Fisher's exact test to evaluate significant differences between pairs of findings. The Kaplan-Meier method and the log-rank test were used for univariate survival analysis. For multivariate survival analysis, each clinicopathologic parameter was analyzed using a Cox proportional hazards regression model. Differences with a *P *< 0.05 were considered statistically significant. All statistical examinations were performed using SPSS 16.0 software package (SPSS Japan, Inc., Tokyo, Japan).

## Results

### Clinical factors

**Figure 1:**
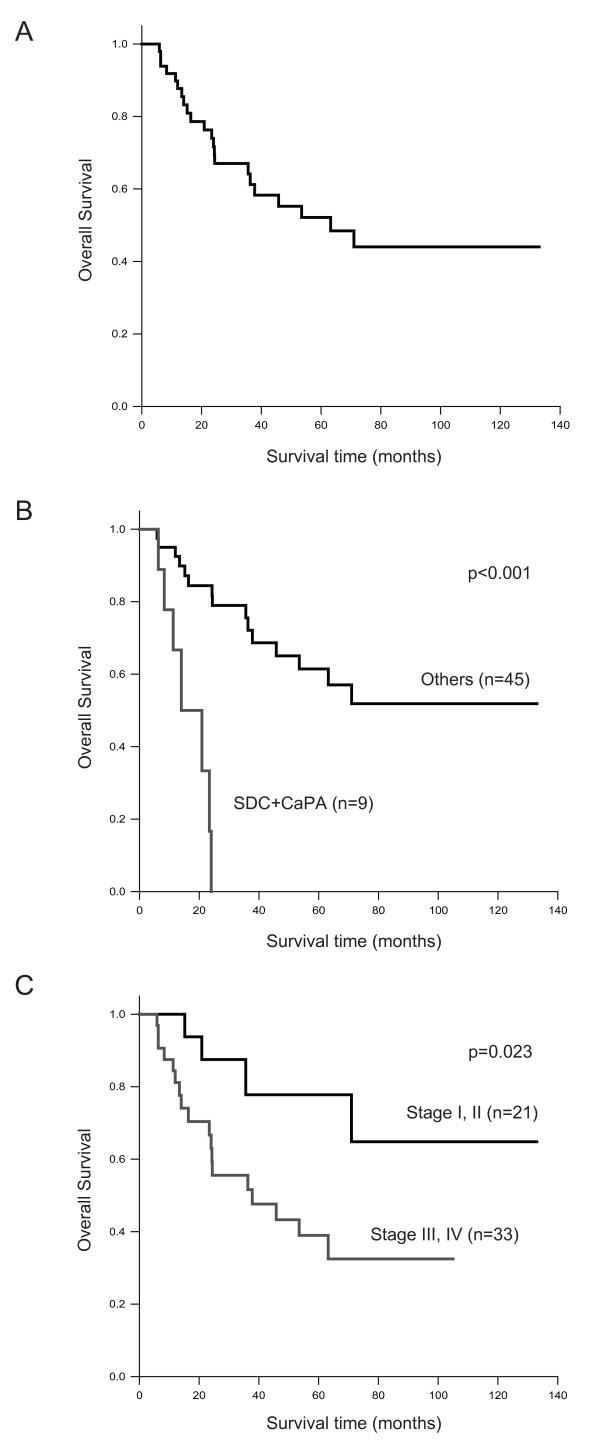
**Kaplan-Meier survival analysis of 54 cases with salivary gland carcinoma**. Overall survival rate for: **(A) **total number of cases;**(B) **histological subtype of tumor comparing salivary duct carcinoma (SDC) and carcinoma ex pleomorphic adenoma (CaPA) with other types; and **(C) **each clinical stage.

Of the 54 patients with salivary gland carcinomas, 37 (69%) were males and 17 (31%) were females. Their age ranged from 6 to 84 years (mean, 61.9 years); patients with mucoepidermoid carcinoma were the youngest (mean, 51.0 years), and patients with salivary duct carcinoma were the oldest (mean, 72.8 years). Most tumors were located in the parotid gland (65%, 35/54), followed by the minor salivary glands (22%, 12/54) and the submandibular gland (13%, 7/54). Of the 54 patients, 21 (39%) presented with stage I or II disease, and 33 (61%) presented with stage III or IV disease. In most cases, the primary treatment was surgical excision (85%, 46/54). The mean follow-up time for all 54 patients was 43 months (range, 6 to 133 months). The overall survival was 55% at 5 years (Fig. 1A). When the survival rate was compared by histologic type, univariate and multivariate survival analyses revealed that the group consisting of salivary duct carcinoma and carcinoma ex pleomorphic adenoma had the shortest survival, less than 3 years (Fig. 1B) (Table 2). In addition, the survival rate of patients with advanced clinical stages (stage III and IV) was significantly shorter than that of patients with stages I and II (p = 0.023) (Fig. 1C).

**Table 2:** Cox regression analysis for survival

Factors	Hazard Ratio (95% confidence interval)	*p*-Value
Age (< 50 vs. > 50)	0.468 (0.100–2.205)	0.337
Gender (male vs. female)	1.403 (0.473–4.159)	0.542
Location (major vs. minor)	0.656 (0.216–1.993)	0.457
Stage (I, II vs. III, IV)	0.424 (0.113–1.592)	0.204
Histological type (SDC+CaPA vs. others)	0.207 (0.056–0.756)	0.017
TopoIIα expression (0, 1+ vs. 2+, 3+)	0.068 (0.018–0.260)	< 0.001

SDC: salivary duct carcinoma; CaPA: carcinoma ex pleomorphic adenoma

### TopoIIα expression in benign and malignant salivary gland tumors

**Figure 2:**
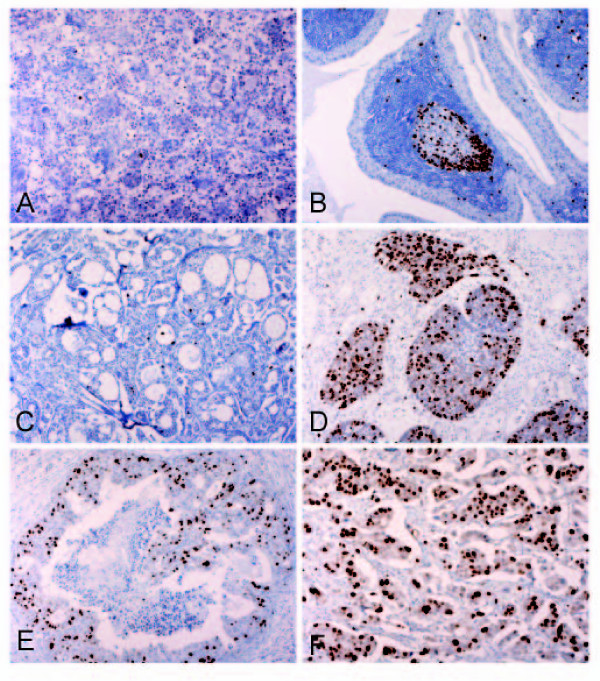
**Immunohistochemistry for topoIIα in salivary gland tumors**. **A: **Pleomorphic adenoma: few positive nuclear expressions in tumor cells (×100). **B: **Warthin's tumor: abundant positive expression in lymphoid cells of germinal centers, not in epithelial tumor cells (×100). **C: **Adenoid cystic carcinoma (Grade 1): low expression in low-grade tubular components (×100). **D: **Adenoid cystic carcinoma (Grade 3): high nuclear expression in high-grade solid areas (×100). **E: **Salivary duct carcinoma: many positive cells in foci with typical comedonecrosis (×100). **F: **Salivary duct carcinoma: strong and diffuse expression observed in the invasive micropapillary variant (×200).

The immunohistochemical expression of topoIIα was negative in the epithelial cells of all 20 benign tumors, including pleomorphic adenoma and Warthin's tumor (Fig. 2A and 2B), whereas positive expression of topoIIα protein (≥10%) was found in 38 of 54 malignant tumors (70%) (p < 0.001) (Table 1). In Warthin's tumors, focal immunopositivity was detected in germinal centers of lymphoid stromal cells, but not in epithelial cells (Fig. 2B). In adenoid cystic carcinoma and mucoepidermoid carcinoma, nuclear staining of topoIIα was positive in 12 cases (71%) and 4 cases (57%), respectively. In adenoid cystic carcinoma, topoIIα expression seemed to be more remarkable in solid type than tumors composed of tubular or cribriform pattern (Fig. 2C and 2D). High expression (3+) was mainly associated with high-grade malignant tumors such as salivary duct carcinoma (83%, 5/6), adenocarcinoma not otherwise specified (43%, 3/7), and carcinoma ex pleomorphic adenoma (100%, 3/3) (Table 1). In salivary duct carcinoma, the nuclear expression of topoIIα was evident in both conventional type with comedonecrosis and microinvasive variant (Fig. 2E and 2F). All malignant components of the 3 carcinomas ex pleomorphic adenomas were mainly composed of salivary duct carcinoma.

### Correlation between TopoIIα expression and prognosis in malignant salivary gland tumors

**Figure 3:**
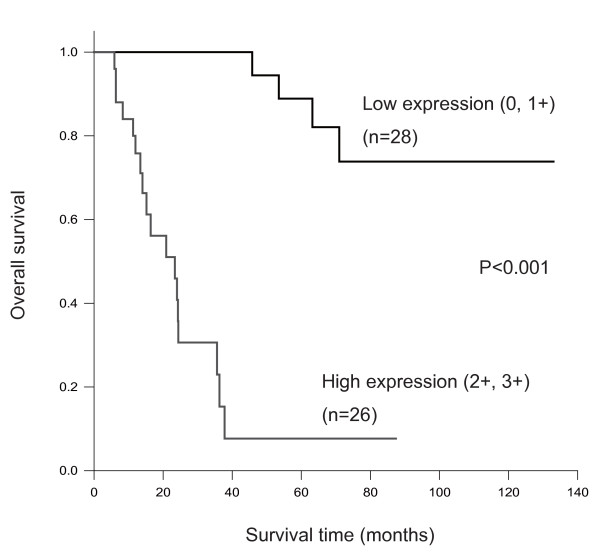
**Kaplan-Meier survival analysis of 54 cases with salivary gland carcinoma by topoIIαgrotein expression level (low vs. high)**.

In order to evaluate the prognostic factors for salivary gland carcinomas, multivariate survival analysis using a Cox proportional hazards regression model was performed, and the expression status of topoIIα protein was identified as the most significant of several clinicopathological factors (p < 0.001) (Table 2). The mortality rate of the low expression group (grade 0 and 1+) was 14.3% (4/28), while that of the high expression group (grade 2+ and 3+) was 69.2% (18/26). The average survival period as determined by the Kaplan-Meier method was 114 months for the low expression group (95% confidence interval: 95–133 months) and 26 months for the high expression group (95% confidence interval: 16–36 months) (Fig. 3); the difference in survival between the groups was statistically significant (p < 0.001).

### Correlation between TopoIIα expression and clinicopathological parameters

The relationship between topoIIα expression and clinicopathological factors is presented in Table 3. TopoIIα overexpression was frequently seen in patients with advanced stage disease (P = 0.024).

**Table 3:** Expression of topoIIα and clinicopathological factors in malignant tumors

Clinicopathologal factors	Low expression (0 and 1+)	High expression (2+ and 3+)	*p*-Value
Age (years)			
< 50	2	5	
> 50	26	21	1.000
*Gender*			
Male	18	19	
Female	10	7	0.565
*Location*			
Major	23	19	
Minor	5	7	0.520
*Stage*			
I, II	16	5	
III, IV	12	21	0.024

### Relationship between TopoIIα expression and histological grade in adenoid cystic carcinoma

Since adenoid cystic carcinoma accounted for the majority of the tumors, the relationship between topoIIα expression and the aggressive histological form was evaluated. The histological grade was determined according to the criteria of Szanto *et al.*: Grade 1, tumor with tubular and cribriform pattern; Grade 2, tumor with a pure cribriform pattern or mixed with less than 30% solid areas; and Grade 3, tumor with more than 30% solid areas [3]. Tumors with grades 1 and 2 predominantly showed low expressions (12/14), whereas all 3 cases with grade 3 tumors demonstrated diffuse and strong expressions (Table 4), though this difference was not statistically significant (p = 1.00).

**Table 4:** Expression status of topoIIα in 17 adenoid cystic carcinomas

		TopoIIα expression	
Histological grade	Number of cases	Low (0 and 1+)	High (2+ and 3+)
Grade 1	n = 7	7	0
Grade 2	n = 7	5	2
Grade 3	n = 3	0	3

## Discussion

The present results show that topoIIα is differentially expressed in salivary gland carcinomas and is associated with aggressive histologic type, advanced disease, and poor clinical outcome. To the best of our knowledge, this is the first study to correlate clinical and prognostic features with topoIIα expression in various types of salivary gland tumors. In a multifactorial analysis, topoIIα expression was more significantly correlated with overall survival than clinical stage (Table 2). Several studies have indicated that high expression of topoIIα is considered a feature of enhanced cellular proliferation [5,6]. It has been reported that overexpression of topoIIα is associated with poorer survival rates in several malignancies, including head and neck squamous cell carcinoma, glioblastoma, and breast cancer [13-15]. The present findings also support an association between topoIIα expression and aggressive histological subtypes (salivary duct carcinoma, carcinoma ex pleomorphic adenoma, adenocarcinoma, high-grade adenoid cystic carcinoma, and carcinosarcoma) [16]. Of 9 cases with salivary duct carcinoma and carcinoma ex pleomorphic adenoma, most (8 cases) showed diffuse and strong nuclear expressions of topoIIα; 2 cases of carcinosarcoma had high expression of topoIIα. Carcinosarcoma, which is composed of both malignant epithelial and malignant mesenchymal components, occasionally occurs from pleomorphic adenoma [17]. Paradoxically, there was no topoIIα expression in benign tumors. In this context, overexpression of topoIIα may be a key event in the malignant transformation of pleomorphic adenoma.

Currently, standard chemotherapy regimens for salivary gland carcinomas have not been established [4]. TopoIIα is not only known to be a prognostic marker, but it is an important target for topoisomerase II poisons, such as anthracycline and epipodophyllotoxin, in several human cancers. These agents exert an anticancer effect by stabilizing the cleavage of double-stranded DNA. Previous investigations have shown that the sensitivity of topoIIα inhibitors, such as anthracyclines or epipodophyllotoxin, is dependent on increased topoIIα expression levels [18,19]. Coincidentally, the gene encoding topoIIα is located at chromosome 17q21-22, which is contiguous with the region encoding HER2. In the field of salivary gland carcinomas, a previous study has shown that overexpression of HER2 is more common in salivary gland carcinomas of excretory cell origin, such as salivary duct carcinoma, mucoepidermoid carcinoma, and squamous cell carcinoma [8]. Nguyen *et al. *[20] also reported a potential association between HER2 overexpression and histological aggressiveness in mucoepidermoid carcinoma. The present data demonstrated that the expression status of topoIIα might be identical to that of HER2, showing common expression in cases of *de novo *salivary duct carcinoma and in malignant components from carcinoma ex pleomorphic adenoma. The molecules associated with amplification of chromosome 17q21-22 may have important roles as diagnostic and therapeutic targets in salivary gland carcinomas. This hypothesis is underscored by a recent phase II trial of herceptin in salivary gland cancers overexpressing HER2. A single dose of herceptin (trastuzumab), a monoclonal antibody directed against HER2 protein, contributed to stabilization of disease in several cases of salivary duct carcinoma [4,8]. Recently, Prat *et al*. [21] reported a metastatic salivary duct carcinoma in which complete response was obtained by the combination of paclitaxel, carboplatin, and herceptin. These observations imply that the therapeutic approaches targeting chromosome17q21-22 region encoding topoisomerase IIα and HER2 proteins may benefit patients with high-grade salivary gland carcinomas.

Adenoid cystic carcinoma is the subtype that has been most widely investigated in various types of salivary gland carcinomas. It has been well known that its clinical behavior is dependent on histological grade, with poorer prognosis in the solid type than in the tubular or cribriform type, and the propensity for perineural invasion is histologically characterized [3]. We previously reported that low expressions of p16 and E-cadherin were associated with poor prognosis and aggressive histological type in adenoid cystic carcinoma [22,23]. In the present study, high expressions of topoIIα were observed in tumors predominantly composed of solid areas, though the difference was not statistically significant due to the small number of cases. Other major proliferation or anti-apoptotic markers, such as Ki-67, bcl-2, and cyclin D1, were frequently expressed in adenoid cystic carcinoma, but they had less of an impact on the discrimination of histological grade of adenoid cystic carcinoma [24-26]. In this context, topoIIα may be an excellent marker to evaluate biological and histological aggressiveness in adenoid cystic carcinoma, although further evaluation in a large subset of tumors is needed.

The present study showed a significant association between topoIIα expression and clinical staging, suggesting that overexpression of topoIIα was associated with rapid growth of cancer cells and susceptibility to locoregional or distant metastasis. High expression of topoIIα is considered to be a general feature of highly proliferating cells with rapid cell cycle acceleration [5,6]. Our observations may be supported by a previous investigation in oral squamous cell carcinoma, which showed that topoIIα expression was more significant in cases with lymph node metastasis than those without lymph node metastasis [27]. Thus, topoIIα may be a valuable marker for evaluating the proliferative activity of salivary gland tumor cells.

## Conclusion

In conclusion, the present study suggests that topoIIα expression is an independent prognostic factor for salivary gland carcinoma, and assessment of topoIIα expression may be valuable for predicting clinical aggressiveness. While certain factors, such as clinical stage and histopathological grade, play a pivotal role in the management of patients with salivary gland carcinoma, high expression of topoIIα may warrant an intensive modality of therapy that includes adjuvant chemotherapy targeting topoIIα protein and radiation. Further investigations are needed to clarify the significance of topoIIα as a practical therapeutic target.

## Competing interests

The authors declare that they have no competing interests.

## Authors' contributions

SM and TS coordinated the study, performed immunohistochemical and statistical analyses, and drafted the manuscript. HK and HM performed histological evaluation of immunostaining. SK and TN contributed to conception and design of the study and interpretation of the data. HS participated in the design and coordination of the study and helped draft the manuscript. All authors have read and approved the final manuscript.

## Pre-publication history

The pre-publication history for this paper can be accessed here:

http://www.biomedcentral.com/1471-2407/9/72/prepub
